# Supplementary material for: MetaQTL: a package of new computational methods for the meta-analysis of QTL mapping experiments
Source: BMC Bioinformatics. 2007 Feb 8;8:49. doi: 10.1186/1471-2105-8-49 (PMC1808479; doi:10.1186/1471-2105-8-49)
Supplement: Additional File 7 — MetaQTL Package : jar file and tutorial. This Zip archive contains both the MetaQTL JAR file and the files of the tutorial. [file 1471-2105-8-49-S7.zip › org.inra.metaqtl/doc/org/thalia/bio/IBioCrossTypes.html]

IBioCrossTypes


|  |  |  |
| --- | --- | --- |
| **Overview** \| **Package** \| **Class** \| **Use** \| **Tree** \| **Deprecated** \| **Index** \| **Help** | |  |
| **PREV CLASS**   **NEXT CLASS** | **FRAMES**    **NO FRAMES**     **All Classes** |  |
| SUMMARY: NESTED \| FIELD \| CONSTR \| METHOD | DETAIL: FIELD \| CONSTR \| METHOD |  |


---


## org.thalia.bio Interface IBioCrossTypes

---

``` public interface IBioCrossTypes ```

Class Description Here

**Author:**
:   Jean-Baptiste Veyrieras

---

| **Field Summary** | |
| --- | --- |
| `static int` | `BC` |
| `static int` | `DH` |
| `static int` | `IRISe` |
| `static int` | `IRISi` |
| `static int` | `RF` |
| `static int` | `RILSe` |
| `static int` | `RILSi` |
| `static int` | `SF` |
| `static int` | `UNKNOWN` |

**Field Detail**

### UNKNOWN

```
static final int UNKNOWN
```

**See Also:**: Constant Field Values

---


### BC

```
static final int BC
```

**See Also:**: Constant Field Values

---


### RILSe

```
static final int RILSe
```

**See Also:**: Constant Field Values

---


### RILSi

```
static final int RILSi
```

**See Also:**: Constant Field Values

---


### SF

```
static final int SF
```

**See Also:**: Constant Field Values

---


### RF

```
static final int RF
```

**See Also:**: Constant Field Values

---


### DH

```
static final int DH
```

**See Also:**: Constant Field Values

---


### IRISe

```
static final int IRISe
```

**See Also:**: Constant Field Values

---


### IRISi

```
static final int IRISi
```

**See Also:**: Constant Field Values


---


|  |  |  |
| --- | --- | --- |
| **Overview** \| **Package** \| **Class** \| **Use** \| **Tree** \| **Deprecated** \| **Index** \| **Help** | |  |
| **PREV CLASS**   **NEXT CLASS** | **FRAMES**    **NO FRAMES**     **All Classes** |  |
| SUMMARY: NESTED \| FIELD \| CONSTR \| METHOD | DETAIL: FIELD \| CONSTR \| METHOD |  |


---
